# HPV16 *E6* Oncogene Contributes to Cancer Immune Evasion by Regulating PD-L1 Expression through a miR-143/HIF-1a Pathway

**DOI:** 10.3390/v16010113

**Published:** 2024-01-12

**Authors:** Georgios Konstantopoulos, Danai Leventakou, Despoina-Rozi Saltiel, Efthalia Zervoudi, Eirini Logotheti, Spyros Pettas, Korina Karagianni, Angeliki Daiou, Konstantinos E. Hatzistergos, Dimitra Dafou, Minas Arsenakis, Amanda Psyrri, Christine Kottaridi

**Affiliations:** 1 Department of Genetics, Development and Molecular Biology, School of Biology, Aristotle University of Thessaloniki, 54124 Thessaloniki, Greece; konstanto@bio.auth.gr (G.K.); dessalton@bio.auth.gr (D.-R.S.); eirinilg@bio.auth.gr (E.L.); spyrospg@bio.auth.gr (S.P.); korinagk@bio.auth.gr (K.K.); kchatzistergos@bio.auth.gr (K.E.H.); dafoud@bio.auth.gr (D.D.); arsenaki@bio.auth.gr (M.A.); 2 2nd Department of Pathology, University General Hospital Attikon, School of Medicine, National and Kapodistrian University of Athens, 12462 Athens, Greece; dleventakou@med.uoa.gr; 3 Research Unit—Oncology Unit, University General Hospital Attikon, School of Medicine, National and Kapodistrian University of Athens, 12462 Athens, Greece; t.zervoudi@hotmail.com; 4 Section of Medical Oncology, Department of Internal Medicine, Attikon University Hospital, Faculty of Medicine, National and Kapodistrian University of Athens, 12462 Athens, Greece

**Keywords:** HPV16, cervical cancer, immune escape, hypoxia, microRNAs, E6, PD-L1, HIF-1a, miR-143

## Abstract

Human Papillomaviruses have been associated with the occurrence of cervical cancer, the fourth most common cancer that affects women globally, while 70% of cases are caused by infection with the high-risk types HPV16 and HPV18. The integration of these viruses’ oncogenes *E6* and *E7* into the host’s genome affects a multitude of cellular functions and alters the expression of molecules. The aim of this study was to investigate how these oncogenes contribute to the expression of immune system control molecules, using cell lines with integrated HPV16 genome, before and after knocking out *E6* viral gene using the CRISPR/Cas9 system, delivered with a lentiviral vector. The molecules studied are the T-cell inactivating protein PD-L1, its transcription factor HIF-1a and the latter’s negative regulator, miR-143. According to our results, in the E6 knock out (E6KO) cell lines an increased expression of miR-143 was recorded, while a decrease in the expression of HIF-1a and PD-L1 was exhibited. These findings indicate that E6 protein probably plays a significant role in enabling cervical cancer cells to evade the immune system, while we propose a molecular pathway in cervical cancer, where PD-L1’s expression is regulated by E6 protein through a miR-143/HIF-1a axis.

## 1. Introduction

Cervical cancer is the fourth most common type of cancer among women worldwide, while more than 95% of total cases are linked to infection with the Human Papillomavirus (HPV) [1,2]. In 2020, there were 604,000 cases of cervical cancer, 342,000 of which led to death [2]. The vast majority of these cases were limited to low- and middle-income countries, considering the lack of prophylactic vaccines and screening methods that are widely used in high-income countries and serve as means for the prevention and early diagnosis of the disease [1,3].

Human Papillomaviruses can be classified according to their ability to either cause benign lesions (low-risk, LR) or lead to several types of cancer (high-risk, HR) [4]. Two of these, HPV16 and HPV18, are responsible for most HPV-related cancers and are the causative agent for approximately 70% of all cervical cancer cases, while HPV16 by its own is responsible for 55% of all cases [4,5].

The HPV genome is a small circular double-stranded DNA of almost 8 kb and it is segmented into three regions: the early region (E) that encodes proteins E1, E2, E4, E5, E6 and E7, the late region (L) which encodes proteins—and L2, and the long control region (LCR) also known as non-coding region (NCR) or upstream regulatory region (URR) [6,7]. The early proteins are necessary for genome replication and transcription, with E5, E6, and E7 being responsible for oncogenesis [4,6]. *L1* and *L2* are important for the viral structure, given that they code the major and minor capsid proteins, respectively, whereas the LCR region does not code any proteins but contains various binding sites for transcription factors [6].

During HPV infection, the viral genome that naturally exists in a circular episomal form can break and integrate into the host’s genome, an event that aids cancer progression. The most common site of integration has been reported to be the E2 ORF, resulting in the loss of the E2 protein, which normally negatively regulates the E6 and E7 oncoproteins [7,8]. Additionally, throughout persistent HPV infection, multiple HPV genes can be expressed by a single strand as a polycistronic pre-mRNA, while several transcripts are produced by alternative splicing, which generates different mRNA expression patterns. Alternative splicing within E6–E7 ORFs is a really common for HR-HPVs, in contrast to LR-HPVs where no splicing in this region has been noticed [9]. Apart from the full-length E6, which is produced from mRNAs with no splicing within E6 ORF, alternative splicing can generate several transcripts containing E6 truncated mRNAs, named E6*I, E6*II, and E6^E7, which are derived from a donor splicing site within the E6 ORF and one of the various acceptor sites located in the early mRNA [9,10]. Protein products generated from the HPV16 E6*I and E6*II transcripts are quite similar. However, it is suggested that they might play different cellular roles, enriching the ways HPV16 can dysregulate the host’s molecular networks and pathways [10].

E6 and E7 activation plays a pivotal role in the development of cancer, considering these proteins’ ability to interfere with many cellular pathways, but most importantly by dysregulating cell cycle, proliferation and apoptosis [6,11]. The way to achieve that is with the well-established process of ubiquitin-dependent proteasome degradation of p53—which regulates apoptosis—by E6 and the inhibition of the retinoblastoma protein (pRb) —which promotes cell cycle progression—by E7. However, E6 seems to participate in another crucial part of malignancy progression, the evasion of host’s immune system [11,12].

HPV is considered a successful pathogen as it has the ability to evade host immune responses and establish long-term persistent infection. The immune checkpoints are critical to maintain tolerance against autoimmunity in physiologic conditions [13]. Programmed cell death protein 1 (PD-1) is a transmembrane protein that acts as a checkpoint molecule on T cells and is overexpressed in the tumor environment [13,14]. Its ligand, programmed death-ligand 1 (PD-L1), is a critical immune checkpoint molecule that has also been observed to be overexpressed on several types of tumors, and cervical cancer appears to be no exception [15,16]. PD-L1 (encoded by *CD274*) is a transmembrane protein synthesized in the endoplasmic reticulum of tumor cells and its interaction with the programmed cell death protein 1 on T-cells restrains antitumor immunity by T-cell activation inhibition or apoptosis, thus leading to cancer’s immune evasion [15,17]. This information renders PD-L1 a possible candidate as a biomarker for cancer prognosis as well as a target for cancer treatment [14,16]. PD-L1 has also been reported to be upregulated under hypoxic conditions, a significant characteristic of tumor microenvironment [18,19].

HIF-1α is a transcription factor that plays a central role in the response to low oxygen levels, or hypoxia, within the tumor microenvironment [20]. HIF-1α’s primary function is to enable cells to adapt to low oxygen, making it a critical player in the survival of both normal and cancer cells [19]. The interaction between PD-L1 and HIF-1α is complex. Under conditions of hypoxia, HIF-1α can upregulate the expression of PD-L1 in cancer cells. This means that in the hypoxic regions of tumors, where immune cells often struggle to function due to low oxygen levels, cancer cells can increase their expression of PD-L1 [18,21,22]. Consequently, this makes the tumor microenvironment even more inhospitable to immune cells, allowing the cancer to evade detection and destruction by the immune system [21,22].

MicroRNAs are small non-coding RNA molecules of 19–25 nucleotides that target and regulate a multitude of mRNAs [23]. The observation that these molecules’ expression is differentiated in several diseases, including cancer, has put a magnifying lens on them as possible biomarkers or therapeutic targets [24,25]. As far as HPV-related cervical cancer is concerned, it has been reported that HPV’s oncoproteins, E6 and E7, seem to target and modify the expression of a plethora of miRNAs, consequently intervening in cellular pathways [23]. HIF-1a appears to be targeted by various microRNAs, one of which is miR-143 that has been reported to negatively regulate the expression of HIF-1a [20,26,27]. Finally, studies on miR-143 have shown the molecule’s aberrant expression in different types of cancer, with the example of cervical cancer, where miR-143 is down-regulated [24,27].

The aim of the present study is to investigate whether HPV16 E6 oncoprotein plays an interplay with a host’s critical immune checkpoint molecule, as well as a known transcription factor that regulates its expression. Undertaking this endeavor, we are committed to understanding the mechanism behind this interaction, with the exploration of a network or a possible molecular pathway that includes HPV16 E6, a miRNA, HIF-1a and PD-L1 that probably promotes cervical cancer’s immune evasion.

## 2. Materials and Methods

### 2.1. Cell Cultures

SiHa, CaSki and HEK293T cell lines were purchased from the American Type Culture Collection (ATCC) (Manassas, VA, USA) and grown in Dulbecco’s Modified Eagle medium (DMEM, Biowest #L0103) supplemented with 10% fetal bovine serum (FBS, Biowest #S1810) (Gibco/Invitrogen; Thermo Fisher Scientific, Inc., Waltham, MA, USA), 1% L-Glutamine (Biowest #X0550) and 1% Penicillin-Streptomycin (Biowest #L0022). According to the ATCC, SiHa cells contain 1–2 copies of the HPV16 genome per cell, whereas CaSki cells contain approximately 600 copies per cell. HCK1T cervical keratinocytes were purchased from Dr Tohru Kiyono of the National Cancer Center Research Institute (Chuo-ku, Tokyo, Japan) [28] to be used as a control and were cultured in KSFM (Keratinocyte serum-free medium, Gibco, #17005042) supplemented with 25 mg Bovine Pituitary Extract (BPE), 2.5 µg EGF, 1% L-Glutamine and 1% Penicillin-Streptomycin as proposed [29]. All cell lines were cultured in a humidified incubator at 37 °C with 5% CO_2_. Wild type cells were harvested after reaching approximately 80% confluence in 100 mm cell culture dishes.

### 2.2. Lentivirus Construction and Cell Transfection

The CRISPRdirect online tool (https://crispr.dbcls.jp/ accessed on 10 January 2023) was used to design HPV16 E6 specific sgRNAs that include the PAM sequence, necessary for the recognition from the CRISPR-Cas9 system. Several proposed sgRNAs were tested in order to opt for the sgRNA set that knocks *E6* out sufficiently. The sgRNAs we ended up using were 16E6T2A (5′-CACCGTCCATAATATAAGGGGTCGG-3′) and 16E6T2B (5′-AAACCCGACCCCTTATATTATGGAC-3′) and they were used for ligation into the lentiCRISPRv2 plasmid (Addgene #52961) which was digested with the BsmBI-v2 digestion enzyme (New England Biolabs, #R0739). The ligated plasmid was cloned into TOP10 competent cells via heat shock and grown on LB agar plates supplemented with ampicillin (100 μg/mL). Colonies were subjected to colony PCR to ensure successful insertion of the sgRNAs, using the universal primer hU6-F (5′-GAGGGCCTATTTCCCATGATT-3′) as a forward primer and as a reverse primer, the reverse sgRNA of each pair. Positive colonies were grown overnight in LB broth supplemented with ampicillin (100 μg/mL). The plasmid was extracted using the MACHEREY-NAGEL Plasmid DNA purification NucleoBond Xtra Maxi (#740414) and was used for transfection of HEK293T cells, using the calcium phosphate method, with the packaging and envelope plasmids psPAX2 (Addgene #12260) and pMD2.G (Addgene #12259). The produced lentivirus was isolated 3 days later by penetrating the media of the transfected cells through a 0.45 mm filter and was used for the transduction of SiHa and CaSki cells which have the HPV16 genome integrated, aiming to knock-out the *E6* oncogene. Optimal lentivirus concentration was determined by using various concentrations of a lentiviral system expressing the green fluorescent protein (GFP). Transfected cells were selected with puromycin (4 μg/mL). In order to have sufficient RNA and protein to extract from the KO cells and to avoid extensive cell-death, transfection was performed in ~80% confluent cells in 100 mm culture dishes and the cells were harvested 4 days post-transfection. Concurrently, we conducted an experiment in which we possessed 3 flasks of the same cell line: one control (untreated cells), one transfected flask (harvested 4 days after puromycin treatment which is the flask used for in vitro experiments) and one flask as apoptosis «observatory», in order to evaluate both changes to cell morphology and decrease in number of viable cells. Successful *E6* knock-out was confirmed by checking p53 protein levels.

### 2.3. Western Blot

Protein was extracted from harvested cells using an RIPA buffer (Cell Signaling, #9806) supplemented with PMSF (Cell Signaling, #8553) and protein concentration was calculated with Bradford assay. Total protein extracts were subjected to electrophoresis on 10% SDS-polyacrylamide gels and transferred on PVDF membranes. After blocking with 5% non-fat milk in TBS-T, the membranes were probed overnight at 4 °C with the following antibodies diluted in the same blocking buffer: anti-p53 (1:2000, mouse, Dako, #M7001), anti-HIF-1a (1:1000, rabbit, Cell Signaling Technology, #36169), anti-PD-L1 (1:500 mouse, Origene, #TA808771), and anti-β-actin (1:2000, mouse, Cell Signaling Technology, #3700). Then, the membranes were washed with TBS-T and incubated for 1 h at room temperature with the following species-specific HRP-linked secondary antibodies: anti-mouse (1:4000 Cell Signaling Technology, #7076) and anti-rabbit (1: 4000 Cell Signaling Technology, #7074). After brief washing with TBS-T, membranes were incubated with LumiGLO chemiluminescent substrate and hydrogen peroxide (Cell Signaling Technology, #7003) and pictures were captured using the Invitrogen iBright FL1500 Imaging System.

### 2.4. qPCR

Harvested cells were subjected to RNA extraction using the TRIzol™ Reagent. Then, the RNA was used for cDNA synthesis, using both SuperScript III Reverse Transcriptase (Invitrogen, #18080) and Mir-X™ miRNA First Strand Synthesis Kit (Takara, #638315). The cDNA was used as a template for qPCR, for which we selected the following primer sets: HIF-1aF (5′-TCTCCATCT-CCTACCCACATACA-3′) with HIF-1aR (5′-TGCTCTGTTTGGTGAGGCTGT-3′), PD-L1F (5′-TATGGTGGTGCCGACTACAA-3′) with PD-L1R (5′-TGGCTCCCAGAATTACCAAG-3′), and GUSBF (5′-CTCATTTGGAATTTTGCCGATT-3′) with GUSBR (5′-CCGAGTGAAGATCCCCTTTTTA-3′). GUSB was used as the internal control for comparative CT analysis. For miR-143 detection, a miR-143 forward primer was designed using the miRprimer2 software version 2.0 (5′-TGCAGTGCTGCATCTCT-3′) and a universal miRNA reverse primer was used that was supplied from the Mir-X™ miRNA First Strand Synthesis Kit. The same kit provided us with a U6 forward and reverse primer set to use as the internal control for comparative CT analysis. The mastermix used was Xpert Fast SYBR (GRiSP, #GE20). Samples were run in an Applied Biosystems StepOnePlus™ Real-Time PCR System and results were received through the StepOne Software v2.3.

### 2.5. Statistical Analysis

Western blot images were quantified using ImageJ (v1.54g) and protein expression was normalized to β-actin expression levels. qPCR results were analyzed using the ΔΔCt method on Microsoft Excel for Microsoft 365 MSO (Version 2309). All experiments were conducted in triplicates. The graphs were created in GraphPad Prism 8 and presented as mean with SD. Finally, an unpaired t-test was applied to examine our results, considering a *p* value ≤ 0.05 to be statistically significant.

## 3. Results

### 3.1. p53 Protein Levels as a Marker for Successful E6 Knock-Out

**Figure 1:**
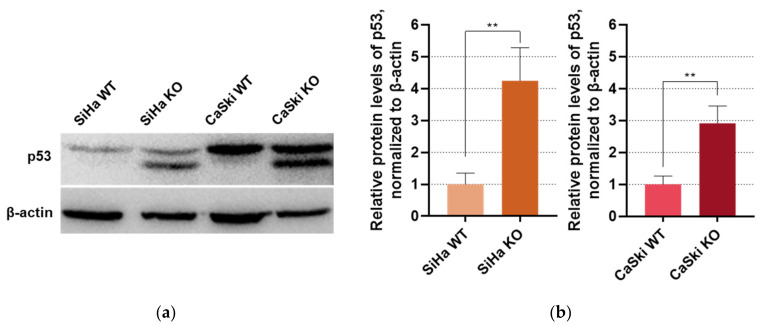
(**a**) Western blot for p53 in SiHa and CaSki cell lines, before and after E6 knock-out. β-actin served as the internal control; (**b**) Bar graphs depicting p53 levels in the cell protein extracts. ** *p* ≤ 0.01.

To confirm the successful knock-out of the *E6* oncogene in the transfected cell lines that carry the HPV16 genome, we determined p53 protein levels via Western blot, taking into consideration the fact that *E6* integration and activation into the host’s genome leads to p53 degradation and contributes to the establishment of the proliferative profile of cancer. As it is presented in Figure 1, the E6KO SiHa and CaSki cell lines exhibit a statistically significant rise in the p53 protein levels when compared to the wild type (WT) control cells. To be precise, in the E6 knock-out cell lines, a second band appears in the blots which can be attributed to the presence of the Δ40p53 isoform of the p53 protein that is mainly targeted and degraded by the E6*II splice variant of E6 [10]. These results show that p53 degradation is reduced in the E6KO cell lines, meaning that *E6* is sufficiently silenced.

### 3.2. E6KO in Cervical Cancer Cell Lines Downregulates PD-L1 Expression

**Figure 2:**
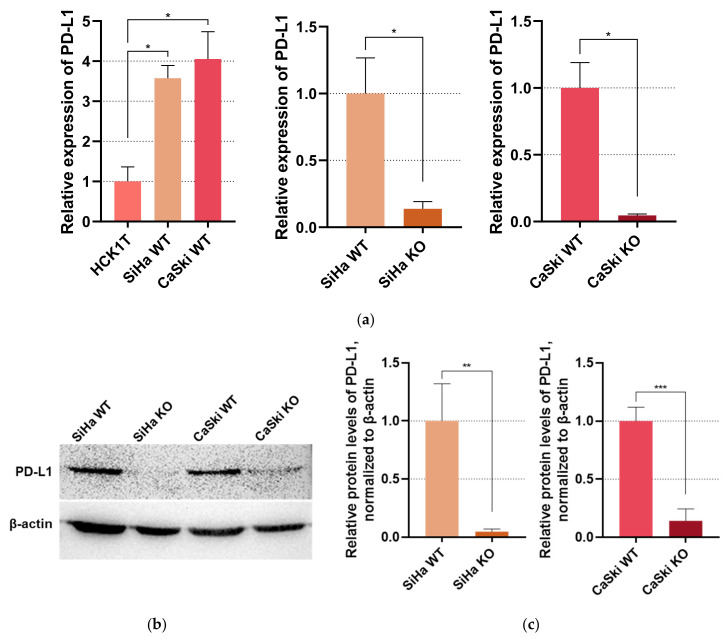
(**a**) Bar graphs representing qPCR results. WT cervical cancer cell lines SiHa and CaSki were compared to normal cervical keratinocytes HCK1T, while E6KO SiHa and CaSki were compared to the WT strains. PD-L1 expression was normalized with GUSB; (**b**) Western blot for PD-L1 in SiHa and CaSki cell lines, before and after *E6* knock-out. Β-actin served as the internal control; (**c**) Bar graphs depicting PD-L1 levels in the cell protein extracts. * *p* ≤ 0.05, ** *p* ≤ 0.01, *** *p* ≤ 0.001.

Firstly, the mRNA levels of PD-L1 were calculated via qPCR to determine whether PD-L1’s gene expression levels are upregulated in SiHa and CaSki wild type cell lines in comparison with the normal cervical keratinocytes HCK1T. As shown in Figure 2a, we indeed noticed a more than threefold increase in PD-L1’s transcript levels in the cancer cell lines. However, PD-L1 is significantly downregulated in the E6KO cell lines. This was further confirmed with Western blot targeting PD-L1 in protein extracts derived from SiHa and CaSki cells before and after E6KO, shown in Figure 2b,c. Additionally, PD-L1 levels of E6KO cells were compared to HCK1T cells and displayed non-significant differences (Figure S1). These results suggest that PD-L1 overexpression in HPV16 positive cervical cancer is related to the activation of E6 in the tumor microenvironment.

### 3.3. HIF-1a Expression Is Controlled by E6 in Cervical Cancer

**Figure 3:**
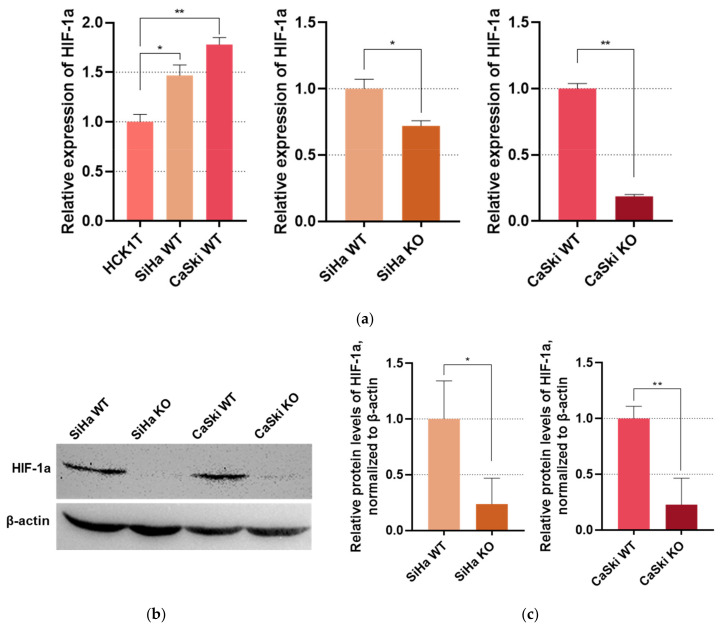
(**a**) Bar graphs representing qPCR results. WT cervical cancer cell lines SiHa and CaSki were compared to normal cervical keratinocytes HCK1T, while E6KO SiHa and CaSki were compared to the WT strains. HIF-1a expression was normalized with GUSB; (**b**) Western blot for HIF-1a in SiHa and CaSki cell lines, before and after *E6* knock-out. β-actin served as the internal control; (**c**) Bar graphs depicting HIF-1a levels in the cell protein extracts. * *p* ≤ 0.05, ** *p* ≤ 0.01.

In order to assess HIF-1a expression, we conducted qPCR on the cDNAs created by the cell lines’ RNA extracts. As it can be observed in Figure 3a, hypoxia-inducible factor-1a is upregulated in cervical cancer cells in comparison to the normal cervical cells. Conversely, the knock-out of *E6* appears to result in a decrease in the transcript levels of HIF-1a, proposing that the aforementioned upregulation is an aftereffect of the E6 activation in HPV16 derived cancer. Moreover, after knocking *E6* out, HIF-1a levels seem to be similar to the normal cervical keratinocytes (Figure S2). Additionally, as shown in Figure 3b,c, HIF-1a protein levels seem to significantly drop in the KO cell lines, further supporting this hypothesis.

### 3.4. E6 Inactivation Increases miR-143 Levels

**Figure 4:**
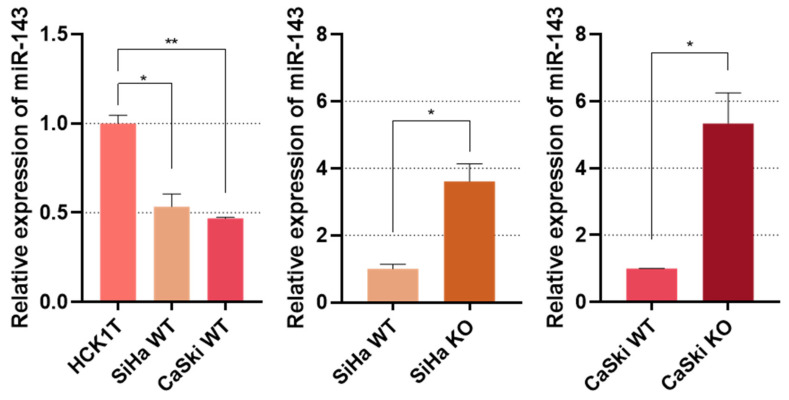
Bar graphs representing qPCR results. WT cervical cancer cell lines SiHa and CaSki were compared to normal cervical keratinocytes HCK1T, while E6KO SiHa and CaSki were compared to the WT strains. miR-143 expression was normalized with U6. * *p* ≤ 0.05, ** *p* ≤ 0.01.

Lastly, we needed to evaluate microRNA-143 expression levels in the cell extracts. To this end, we conducted qPCR using microRNA-specific cDNA libraries of the cell lines and the results we received are presented in Figure 4. miR-143 seems to be downregulated in WT SiHa and CaSki cells when compared to the HCK1T keratinocytes. Nonetheless, miR-143 is considerably upregulated in the cancer cell lines subjected to *E6* knock-out and does not show significant difference in expression when compared to the HCK1T cells (Figure S3).

## 4. Discussion

Cervical cancer is the most common HPV-related disease with an overall 5-year relative survival rate of 67% [2,30]. Patients diagnosed with cervical cancer may be subjected to a variety of treatment schemes consisting of radiation therapy, chemotherapy and immunotherapy, to name but a few [30]. An immunotherapy drug that has shown potential in increasing survival rate to patients with different types of cervical cancer is Pembrolizumab, a monoclonal antibody that targets and binds on the PD-1 protein on the surface of T-cells, thus denying PD-1/PD-L1 interaction and allowing T-cell mediated destruction of cancer cells [31,32]. Several studies have reported that a great number of cervical carcinomas overexpress PD-L1 [15,16,31,32], findings that go in line with the results of our study, where we showed upregulated expression of the specific molecule in cervical cancer cell lines that carry the HPV16 genome. So, our initial scientific question was the possible role of viral oncogenes and specifically of *E6*, in pathways that take part in PD-L1 regulation, to answer this question regarding the knocking-out of *E6* oncogene constituted our strategy. Definitely, we strongly kept in mind that *E6* knockouts lead to apoptosis and cell death of HPV containing cell lines [33]. Provided that for the apoptotic response 4–6 days are needed in order to notice any difference in cell viability [34], cells in our experimental model were harvested 4 days post-transfection while an additional flask with *E6* KO cells evaluated in parallel both for changes to cell morphology and decrease in number of viable cells.

As far as HPV16-related cervical cancer is concerned, our results indicate that the E6 oncoprotein seems to contribute to the PD-L1 overexpression, considering that PD-L1 levels drop significantly in the cell lines that have gone through E6 knock-out. This implies that *E6* integration in the host’s genome triggers a number of alterations that ultimately lead to cancer cells escaping immunosurveillance. In order to unravel the mystery of which molecules take part in this event, it was important to examine PD-L1’s transcription factors.

Hypoxia-inducible factor-1a is an important transcription factor that has been proven to act as a master regulator of quite a few genes, especially in the hypoxic tumor microenvironment where HIF-1a thrives. In a normal aerobic environment, HIF-1a is regulated by the ubiquitin-protease system, so its expression in normal cells is not stable. On the contrary, in the tumor microenvironment, hypoxia leads to the continuous accumulation of HIF-1α, making cancer cells continuously adapt to hypoxia [35]. HIF-1a is in an interplay with all hallmarks of cancer, like genomic instability, inflammation, vascularization, tumor invasion and survival, amongst others [36,37]. Furthermore, there is strong evidence that HIF-1a reinforces cancer’s immune evasion by dysregulating various characteristic mechanisms of immune response, like the production of cytokines, assisting the activity of immunosuppressive M2 macrophages in addition to inducing the expression of immune checkpoint inhibitors [38,39]. PD-L1 is one of these immune checkpoint molecules that fall under the regulatory control of HIF-1a.

Studies in the past have showcased HIF-1a’s ability to positively regulate PD-L1 under hypoxic conditions, as well as HPV16 E6’s upregulation of HIF-1a in the tumor microenvironment [40,41]. However, the molecular mechanism of the interaction between HIF-1a and PD-L1 in the presence of HPV16 in cervical cancer has not been studied. Our research strongly supports the existing data, given that HIF-1a mRNA levels appear significantly increased in the cervical cancer cell lines when compared to the normal cervical keratinocytes, and correlates E6’s presence in the cancer cells with HIF-1a’s expression increase. To elaborate, knocking *E6* out considerably decreases HIF-1a’s levels, a change that, in combination with PD-L1’s decrease in the E6KO cell lines, implicates that PD-L1’s upregulation in HPV16-caused cervical cancer happens in a HIF-1a related manner.

An additional effect of the presence and accumulation of HIF-1a in tumors is presumed to be the contribution to tumor chemoresistance by obstructing drug transport and uptake, whereas its overexpression is linked to worse prognosis for patients with cervical cancer [39,42]. On the other hand, a molecule that has given signs of decreasing cancer cell resistance to chemotherapeutics in addition to inhibiting several of the hallmarks of cancer, like proliferation, tumor invasion and metastasis, is microRNA-143 [43,44,45,46].

MicroRNAs regulate a wide range of biological processes in the host-pathogen interactions and are commonly encoded by viruses that undergo long-term persistent infection, including HPV, acting either as oncogenes or as tumor suppressors by targeting different mRNAs. Dysregulation of miRNA expression causes abnormal cell growth and differentiation, assisting to the development of cancer or other diseases [24]. The expression of several miRNAs in cervical cancer has been studied in order to further understand the way the Human Papillomaviruses interact with the host’s cellular pathways, and this research still has a lot to unravel. MicroRNAs’ expression profiles seem to differ depending on the tissue studied and the cancer differentiation state, making the classification of each biomolecule difficult. MicroRNA-143 has been reported to be up- or downregulated in the tumor microenvironment of different tissues [47]. However, when it comes to cervical cancer, many studies agree on miR-143’s down-regulation throughout all the stages of cancer progression when compared to healthy tissue [23,24,27,47]. Our study’s findings are consistent with this data, given that miR-143 levels are decreased in SiHa and CaSki cell lines in comparison with the HCK1T normal cervical keratinocytes.

Induced expression of miR-143 in cervical cancer cells resulted into increased apoptosis as well as improved reaction to Cisplatin treatment, data that renders miR-143 a possible tumor-suppressing microRNA, as well as a candidate biomarker for prognosis or the optimization of cervical cancer treatment [27,44] Zhao et al. demonstrated, using dual-luciferase reporter gene assay, that miR-143 directly targets and silences HIF-1a by binding on its 3′UTR, thus, negating the transcription factor’s effects in the cervical tumor microenvironment and ameliorating the cancerous phenotype. As mentioned above, in our experiments, miR-143 expression drops in the cells that carry the HPV16 genome, however, this downregulation seems to reverse after the knock-out of *E6* in the same cell lines, where a significant increase in the specific microRNA’s levels is exhibited. These results suggest that the expression of miR-143 is regulated by the activation of E6 after its integration into the host’s genome.

miR-143 is definitely not the first case of a microRNA being regulated by E6 to promote immune escape of cervical cancer by targeting PD-L1. As Ling et al. have already shown, another miRNA—the miR-142-5p, seems to follow the same expression pattern of miR-143 in cervical cancer, whereas when overexpressed it appears to directly interact with PD-L1 and negatively regulate its expression [48]. Likewise, our results indicate that miR-143 is in an interplay with the expression of PD-L1 in cervical cancer cells, yet this interaction possibly happens in an indirect way, via the HIF-1a inhibition. However, regardless of the way these miRNAs act on the immune checkpoint molecule, they both seem to be targets of the HPV16’s E6 oncoprotein.

The way E6 acts on miR-143 is yet to be investigated. A hypothetical way in which E6 affects miR-143’s expression could be via the p53 degradation, for there is evidence that supports that p53 post-transcriptionally upregulates miR-143’s expression [49,50]. This interaction could explain how *E6* integration into the host’s genome results in miR-143’s negative regulation, since p53 gets degraded by E6 and can no longer enhance miR-143’s expression. Nonetheless, the scenario that E6 directly targets miR-143 in either stage of the microRNA’s biogenesis and maturation cannot be ruled out, but more research needs to be conducted. Future experiments, including transfections with synthetic circRNA sponges, represent a strategy for achieving targeted loss of miR143 function where the link between E6–p53–miR143–HIF-1a–PD-L1 would be further enlightened.

## 5. Conclusions

In order to enhance PD-L1 targeting in cervical cancer treatment and improve response rate, it is crucial to further investigate the molecules and the pathways that take part in PD-L1 regulation. Towards this direction, our endeavor shows our preliminary data regarding a mechanistic pathway were HPV16 viral oncogenes have a possible role. Our data could propose a molecular network that leads to PD-L1’s upregulation in HPV16 related cervical cancer through a mir-143/HIF-1a mediated axis and, consequently, to cervical cancer’s ability to escape immunosurveillance.

## Data Availability

Data are contained within the article and Supplementary Materials.

## Supplementary Materials

The following supporting information can be downloaded at: https://www.mdpi.com/article/10.3390/v16010113/s1, Figure S1: Bar graphs representing qPCR results. KO cervical cancer cell lines SiHa and CaSki were compared to normal cervical keratinocytes HCK1T. PD-L1 expression was normalized with GUSB. ns = non-significant; Figure S2: Bar graphs representing qPCR results. KO cervical cancer cell lines SiHa and CaSki were compared to normal cervical keratinocytes HCK1T. HIF-1a expression was normalized with GUSB. ns = non-significant; Figure S3: Bar graphs representing qPCR results. KO cervical cancer cell lines SiHa and CaSki were compared to normal cervical keratinocytes HCK1T. miR-143 expression was normalized with U6. ns = non-significant.
