# Correction: Konstantopoulos et al. HPV16 *E6* Oncogene Contributes to Cancer Immune Evasion by Regulating PD-L1 Expression through a miR-143/HIF-1a Pathway. *Viruses* 2024, *16*, 113

**DOI:** 10.3390/v16121817

**Published:** 2024-11-22

**Authors:** Georgios Konstantopoulos, Danai Leventakou, Despoina-Rozi Saltiel, Efthalia Zervoudi, Eirini Logotheti, Spyros Pettas, Korina Karagianni, Angeliki Daiou, Konstantinos E. Hatzistergos, Dimitra Dafou, Minas Arsenakis, Amanda Psyrri, Christine Kottaridi

**Affiliations:** 1 Department of Genetics, Development and Molecular Biology, School of Biology, Aristotle University of Thessaloniki, 54124 Thessaloniki, Greece; konstanto@bio.auth.gr (G.K.); dessalton@bio.auth.gr (D.-R.S.); eirinilg@bio.auth.gr (E.L.); spyrospg@bio.auth.gr (S.P.); korinagk@bio.auth.gr (K.K.); angeldaiou@bio.auth.gr (A.D.); kchatzistergos@bio.auth.gr (K.E.H.); dafoud@bio.auth.gr (D.D.); arsenaki@bio.auth.gr (M.A.); 2 2nd Department of Pathology, University General Hospital Attikon, School of Medicine, National and Kapodistrian University of Athens, 12462 Athens, Greece; dleventakou@med.uoa.gr; 3 Research Unit—Oncology Unit, University General Hospital Attikon, School of Medicine, National and Kapodistrian University of Athens, 12462 Athens, Greece; t.zervoudi@hotmail.com; 4 Section of Medical Oncology, Department of Internal Medicine, Attikon University Hospital, Faculty of Medicine, National and Kapodistrian University of Athens, 12462 Athens, Greece

## Addition of an Author

Amanda Psyrri was not included as an author in the original publication [1]. The corrected Author Contributions statement appears here. The authors state that the scientific conclusions are unaffected. This correction was approved by the Academic Editor. The original publication has also been updated.
