# Supplementary material for: HPV16 E6 Oncogene Contributes to Cancer Immune Evasion by Regulating PD-L1 Expression through a miR-143/HIF-1a Pathway
Source: Viruses. 2024 Jan 12;16(1):113. doi: 10.3390/v16010113 (PMC10819893; doi:10.3390/v16010113)
Supplement: Supplementary file 1 [file viruses-16-00113-s001.zip › viruses-2824509-supplementary.pdf]

Supplementary Figures

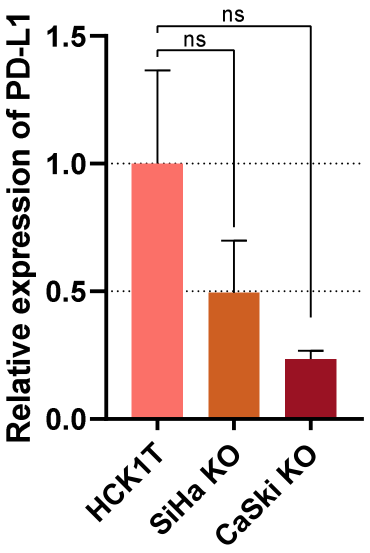

Figure S1. Bar graphs representing qPCR results. KO cervical cancer cell lines SiHa and CaSki were compared to normal cervical keratinocytes HCK1T. PD-L1 expression was normalized with GUSB. ns = non-significant.

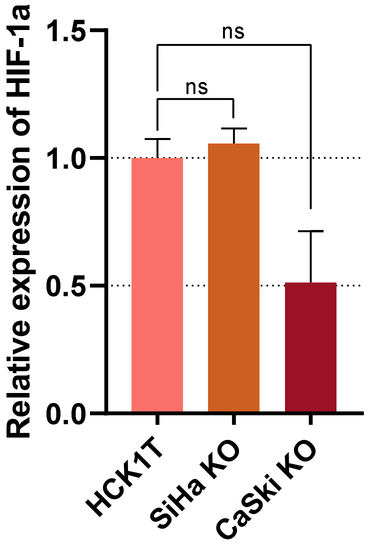

Figure S2. Bar graphs representing qPCR results. KO cervical cancer cell lines SiHa and CaSki were compared to normal cervical keratinocytes HCK1T. HIF-1a expression was normalized with GUSB. ns = non-significant.

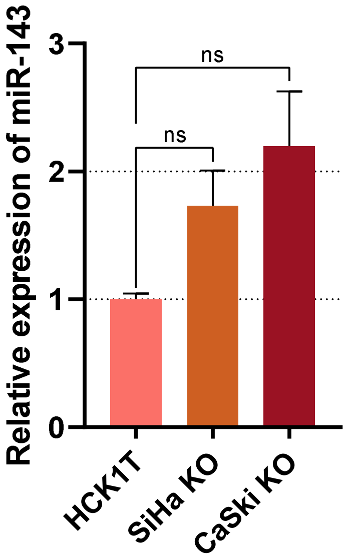

Figure S3. Bar graphs representing qPCR results. KO cervical cancer cell lines SiHa and CaSki were compared to normal cervical keratinocytes HCK1T. miR-143 expression was normalized with U6. ns = non-significant.
